# DNA Methylation Profiles of Vegans and Non-Vegetarians in the Adventist Health Study-2 Cohort

**DOI:** 10.3390/nu12123697

**Published:** 2020-11-30

**Authors:** Fayth L. Miles, Andrew Mashchak, Valery Filippov, Michael J. Orlich, Penelope Duerksen-Hughes, Xin Chen, Charles Wang, Kimberly Siegmund, Gary E. Fraser

**Affiliations:** 1 Adventist Health Study, Loma Linda University, Loma Linda, CA 92350, USA; fmiles@llu.edu (F.L.M.); amashchak@llu.edu (A.M.); morlich@llu.edu (M.J.O.); 2 Center for Nutrition, Healthy Lifestyle, and Disease Prevention, School of Public Health, Loma Linda University, Loma Linda, CA 92350, USA; 3 Department of Preventive Medicine, School of Medicine, Loma Linda University, Loma Linda, CA 92350, USA; 4 Department of Basic Sciences, School of Medicine, Loma Linda University, Loma Linda, CA 92350, USA; vfilippov@llu.edu (V.F.); pdhughes@llu.edu (P.D.-H.); xchen@llu.edu (X.C.); chwang@llu.edu (C.W.); 5 Department of Medicine, School of Medicine, Loma Linda University, Loma Linda, CA 92350, USA; 6 Center for Genomics, School of Medicine, Loma Linda University, Loma Linda, CA 92350, USA; 7 Department of Preventive Medicine, Keck School of Medicine of the University of Southern California, Los Angeles, CA 90033, USA; kims@usc.edu

**Keywords:** DNA methylation, epigenetics, Adventist Health Study-2, vegetarian diet, linear regression, permutation

## Abstract

We sought to determine if DNA methylation patterns differed between vegans and non-vegetarians in the Adventist Health Study-2 cohort. Genome-wide DNA methylation derived from buffy coat was profiled in 62 vegans and 142 non-vegetarians. Using linear regression, methylation of CpG sites and genes was categorized or summarized according to various genic/intergenic regions and CpG island-related regions, as well as the promoter. Methylation of genes was measured as the average methylation of available CpG’s annotated to the nominated region of the respective gene. A permutation method defining the null distribution adapted from Storey et al. was used to adjust for false discovery. Differences in methylation of several CpG sites and genes were detected at a false discovery rate < 0.05 in region-specific and overall analyses. A vegan diet was associated predominantly with hypomethylation of genes, most notably methyltransferase-like 1 (*METTL1*). Although a limited number of differentially methylated features were detected in the current study, the false discovery method revealed that a much larger proportion of differentially methylated genes and sites exist, and could be detected with a larger sample size. Our findings suggest modest differences in DNA methylation in vegans and non-vegetarians, with a much greater number of detectable significant differences expected with a larger sample.

## 1. Introduction

Findings from the Adventist Health Study-2 (AHS-2) cohort have been prominent among epidemiologic studies linking a vegetarian dietary pattern to lower risks of diabetes, metabolic syndrome, and coronary heart disease [1,2,3,4,5] as well as lower risks of gastrointestinal and prostate cancers, among others [6,7,8]. Besides avoiding meat, vegetarians and particularly vegans in this cohort have markedly higher consumption of plant-based foods including fruits, vegetables, whole grains, soy, and nuts, but lower consumption of sweets, snack foods, refined grains, and caloric beverages [9], and healthier profiles of biomarkers of dietary intake, including phytochemicals or bioactive compounds [10] relative to non-vegetarians. However, there is limited understanding of the underlying molecular mechanisms that could help explain the probable ability of vegetarian dietary patterns to prevent chronic diseases, or to buttress causal interpretations.

DNA methylation involves the chemical addition of a methyl group at the 5′ position of a cytosine residue, and may be influenced by diet, among other lifestyle or environmental factors. Such modifications can potentially alter gene expression and the development of chronic, autoimmune, or aging-related diseases [11,12]. Diet may influence gene-specific or global DNA methylation by (1) providing or modifying the availability of methyl donor compounds or cofactors that regulate one- carbon metabolism [13], (2) regulating enzymes that catalyze or reverse methylation, such as DNA methyl transferases (DNMTs) or ten or eleven translocation (TET) enzymes [14,15], and (3) indirectly regulating inflammation or oxidative stress [16]. Vegetarian or plant-based diets are rich in polyphenols and secondary plant metabolites that could inhibit the activity of DNMT to prevent cancer [14,17,18]. On the other hand, diets high in animal-based and fatty foods may generate pro-inflammatory compounds [19,20,21,22] and metabolites [15,23] that alter methylation and increase activity of TET enzymes to promote cancer.

Besides specific dietary components, there is some evidence from animal studies and human intervention trials that dietary conditions such as high fat content or caloric restriction may alter genome-wide and gene-specific DNA methylation [24,25,26,27,28,29,30]. However, it is not clear how habitual dietary patterns differing in consumption of plant- and animal-based foods differ in terms of DNA methylation patterns. The aim of the current study was to examine genome-wide DNA methylation patterns in long-term vegan and non-vegetarian participants of the AHS-2 cohort to identify differentially methylated genes. Our approach involved a covariate-adjusted analysis of methylation of individual CpG sites, as well as of CpGs annotated to their respective genes (gene methylation), considering various gene regions, and employing an adapted Storey et al. [31,32] permutation method to correct for false discovery.

## 2. Materials and Methods

### 2.1. Study Population

Participants in the current study are members of the larger AHS-2 cohort, established in 2002–2007 as a national study of Seventh-day Adventists, about ½ of whom are vegetarian. In this sub-study, 143 participants were Caucasian, and 61 were African-American or Black. Subjects were selected from the AHS-2 biorepository for DNA methylation analyses conducted at three separate times variably balanced by age, sex, diet group, and/or ethnic group. All subjects were pooled for the analyses performed in the current study. Participants were classified a priori into vegetarian diet groups based on their responses to the food frequency questionnaire (FFQ) completed upon enrollment as follows: vegans never or rarely (less than once per month) consumed eggs, dairy, fish and other meats, and non-vegetarians consumed non-fish meats at least once a month and any meat (including but not only fish) more than once per week.

All investigations were carried out following the rules of the Declaration of Helsinki of 1975, revised in 2013. The project was originally approved by the Loma Linda University institutional review board, protocol: 48134.

### 2.2. Laboratory Analysis and Data Processing

Fasting blood samples were collected at field clinics held in church halls, as described previously [33]. Buffy coat was removed and diluted to a final volume of 8 mL with phosphate buffered saline, then aliquoted into straws and frozen at −180 °C in nitrogen vapor for storage in the AHS-2 biorepository. Genomic DNA from buffy coat samples was isolated using the Quick-gDNA MiniPrep kit (Zymo Research, Irvine, CA, USA) according to the manufacturer’s protocol. After bisulfite sequencing, genome-wide methylation analysis was performed using the Infinium MethylationEPIC (*n* = 67) and HumanMethylation450 BeadChip (*n* = 137) arrays at the University of California, Los Angeles Neuroscience Genomics Core. Raw data were summarized into BeadStudio IDAT files for further analysis.

Methylation data were processed using R version 3.6.1. Initial preprocessing and normalization procedures were conducted using the minfi package in R [34]. Raw data generated from methylation analysis were normalized with the single-sample normal-exponential out-of-band (ssNoob) method as recommended by Fortin (2017) for combined 450 k and EPIC datasets. Probes were excluded that were cross-reactive, within 10 bp of single nucleotide polymorphisms (SNPs) [35,36], or present on X/Y chromosomes. Furthermore, CpG probes with bead counts less than 3 in more than 2% of samples or detection *p* values above 0.01 in more than 1% samples were excluded. After these normalization and processing steps, 313,206 CpG sites (common to both BeadChip arrays) remained for analysis.

As an additional layer of control and primarily to adjust for unwanted variation including batch, chip, positional, cell heterogeneity, and study effects, a surrogate variable analysis was used from the SmartSVA R package. With this method, influential principal components of residuals from regressions of CpG methylation on dietary groups are converted into surrogate variables, and subsequently included in final regression models, thereby accounting for unwanted variation from other sources of “error” [37].

### 2.3. Statistical Analyses

DNA methylation β values were obtained from the BeadChip array, representing the methylation frequency within an individual sample (ratio of methylated signal divided by the sum of methylated and unmethylated signals) for each target CpG or gene ranging from 0 (not methylated) to 1 (fully methylated). Subsequently the log2 ratio of the methylated to unmethylated signal was calculated to obtain M values. Using SmartSVA (39), surrogate variables were created from regression residuals conditional on covariates, age (continuous), sex (male, female), ethnic group (Black, Caucasian), and body mass index (BMI; continuous). To examine the association between dietary pattern and DNA methylation, a linear model was then fitted where the response variable was the residual when the M values were regressed on these covariates (except BMI) and the surrogate variables [38]. This represents non-BMI covariate-adjusted methylation levels for individual CpG sites [39], or the average of covariate-adjusted methylation level for CpGs associated with each gene. The independent variable came from residuals when dietary pattern was regressed on these same covariates, this then representing covariate-adjusted dietary pattern. Body mass index was not included as a covariate, given that it may be a mediator in some cases between diet and CpG methylation.

For comparison, separate linear regression models were fitted excluding the SmartSVA method, where models were additionally adjusted for position on the BeadChip (*n* = 8 rows), the original sub-study (*n* = 3) along with array (450 K, 800 K), and cell type heterogeneity (CD8T, CD4T, NK, Bcell Mono, Gran) using the approach of Houseman et al. [38].

CpG sites were associated with genes by calculating an unweighted average of covariate-adjusted M values of all CpG sites in the dataset that were annotated to the gene of interest and in the nominated gene region, where applicable. When analyzing a particular region, genes were only included where at least two CpG sites were available in that region. Thus, besides analyzing methylation of all represented sites, or average methylation across a gene, methylation was also analyzed according to 1) genic/intergenic region (200 or 1500 base pairs upstream of the transcription start site (TSS200 and TSS1500, respectively), the 5′ untranslated region (UTR), first exon, gene body, 3′ UTR, and intergenic regions, 2) in relation to CpG islands (CpG island, north shore, south shore, north shelf, south shelf, and open sea regions), and 3) in the promoter, including CpG islands within the promoter.

An adapted permutation-identified null distribution (Storey et al. method, references 31, 32) was used to adjust for multiple comparisons to avoid the risk of severe false discovery. The measure of DNA methylation differences by diet pattern for each CpG site or gene is the corresponding partial t statistic for each CpG site, and for genes a joint t statistic averaging multivariable-adjusted methylation status across relevant CpGs in a particular gene. The permutation method was used to define the null distribution of the t scores for CpG sites. Then for genes, in separate analyses, all CpG sites in a nominated region relevant to that gene were used in the averaging process, not only the CpG sites that were previously statistically significant. To adjust for covariates when planning a permutation approach, the residual method was used as has been recommended [40], this being applied to both the independent variables aside from diet (the exposure of interest) and the dependent variable as described above. This reduces the independent variables to one for permutation, which already takes account of covariances between the covariates. Covariances between the residualized dependent M values are retained as these dependent variables are not permuted. Recognizing that the real data are a mixture of null and non-null methylation sites/genes, an estimate of the proportion of null features allows a direct estimate of the false discovery rate (FDR) [32], and hence the more useful selection of only those with small FDR. The method also allows an estimate of the number of all differentially methylated sites/genes [31,32]), although only a proportion can be individually identified with acceptable FDR in this relatively small dataset. All statistical analyses were performed with R software.

## 3. Results

### 3.1. Methylation Differences in Vegans and Non-Vegetarians

Demographic characteristics of study participants are shown in Table 1. There were no statistically significant differences in the distribution of males and females, or in age, comparing vegans and non-vegetarians. As expected, vegans had lower BMI relative to non-vegetarians (24.3 vs. 28.0; *p* < 0.001). This was viewed as a potentially mediating variable in statistical analyses.

For this study, we compared methylation of individual CpG sites and genes in vegans relative to non-vegetarians, dividing CpG sites into separate categories defined by gene region, namely, genic/intergenic, relation to CpG islands, and promoter regions.

#### 3.1.1. Differential Methylation of Select Regions Associated with Individual Genes

1. Proportions of Differentially Methylated Gene Regions

**Figure A1:**
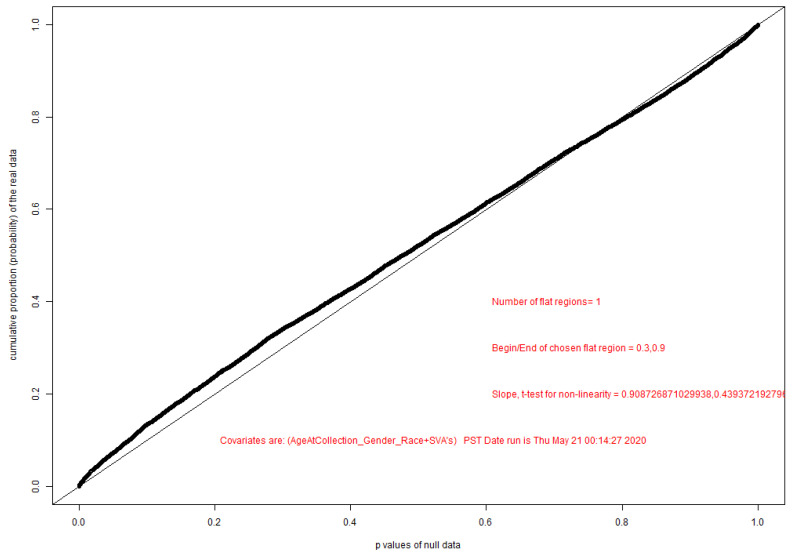
Plot of cumulative *p* values from real data (dark line), and from a hypothetical data where all data are null (fine line). Differential methylation: average of CpG sites in gene body regions, comparing non-vegetarians to vegans.

There were greater proportions of genes with at least two probes in a related CpG island (74%) or TSS1500 (72%) region, followed by those with sites in the gene body (65%) (Table 2). The majority of differentially methylated genes were hypomethylated in vegans. When differential methylation was analyzed after averaging CpG methylation across an entire gene, within each of the 18,627 genes (i.e., labelled “All”) represented on the array and in our analytical dataset, a total of 18 genes (4 hypermethylated, 14 hypomethylated) were found to be differentially methylated after adjustment for false discovery (Table 2). When differential methylation was analyzed according to methylation of genic/intergenic regions, 21 genes (including three identified in the analysis of overall gene methylation, “All”) showed differential methylation in the gene body, representing the most differentially methylated genes for any region. Identifying a central linear region of a plot of cumulative probabilities of the null vs. actual distributions of t scores (adaptation of methods in references [31,32]) revealed that an estimated 1081 (6%) of the 18,627 genes analyzed are non-null (Table 2 and Appendix A), although the majority of these cannot be specifically identified here. The greatest proportion of estimated non-null genes relative to the respective total number of genes for methylation of a given region were genes defined by methylation of the gene body (9%, see Figure A1 of Appendix A), with relatively high proportions also noted when defined by methylation of the TSS1500 (7%), north shelf (7%), and open sea (7%) regions. As only t-scores of non-null sites will systematically increase (in absolute value) as sample sizes increase, it is possible to estimate the effects of larger sample size on the proportion of differentially methylated genes that could then be identified (Appendix A). With increasing sample size, the number of differentially methylated genes comparing vegans with non-vegetarians steadily increases (Table 3). Fewer differentially methylated genes were observed with exclusion of surrogate variables (SmartSVA) from the analytical approach (Supplementary Table S1), although greater numbers of differentially methylated CpG sites than genes (observed and non-null) were noted in analyses excluding surrogate variables (Supplementary Table S2).

2. Unique Genes Showing Differential Methylation

*Methyltransferase-like 1* (*METTL1*) was significantly and consistently hypomethylated in vegans in both analyses of promoter methylation (62,398 CpG sites distributed among 9131 genes) and overall methylation of all genes present on the array and in our analytical set (313,161 CpG sites distributed among 18,627 genes), this with either the inclusion of SmartSVA (Table 4 and Table 5) or the exclusion of surrogate variables from models (Supplementary Tables S3 and S4). Other genes that were significantly hypomethylated in the promoter regions in analyses both with and without surrogate variables included *ribosomal protein L38* (*RPL38*), *snurportin 1* (*SNUPN*), FLVCR heme transporter 2 (*FLVCR2*), and *CKLF-like MARVEL transmembrane domain containing 7* (*CMTM7*). Two genes, *METTL1* and *nei-like DNA glycosylase 2* (*NEIL2*), were significantly hypomethylated in CpG islands within the promoter, in analyses with and without SmartSVA (Table 5 and Supplementary Table S4). Besides *METTL1*, genes showing differential methylation in analyses of both promoter methylation and overall methylation of all genes included *RPL38* (SmartSVA analysis) (Table 4 and Table 5), and *FLVCR2* (analysis excluding SmartSVA) (Supplementary Tables S3 and S4). Differential methylation of *D-dopachrome tautomerase-like* (*DDTL*) and *aryl hydrocarbon receptor nuclear translocator 2* (*ARNT2*) was observed in analyses of genes defined by methylation of the gene body, as well as of all genes (SmartSVA analysis, Table 4), and the same was true for *LIM homeobox protein 3* (*LHX3*) in models excluding surrogate variables (Supplementary Table S3). *Glutathione S-transferase theta pseudogene 1* (*GSTTP1*), detected in analysis of the open sea region showed the most marked hypomethylation in vegans (fold change of 0.72 and 0.76 in models with and without SmartSVA, respectively), whereas NACHT and WD repeat domain containing 2 (NWD2), identified in analysis of methylation of the south shore region, showed the greatest increase in gene methylation (fold change, 1.12) (Table 5 and Supplementary Table S4).

#### 3.1.2. Differential Methylation of CpG Sites

1. Proportions of Differentially Methylated CpG Sites by Region

Considering CpG sites, the majority of CpG sites are mapped to the gene body (36%), open sea (34%), and island (32%) regions (Table 6). A large proportion of the promoter region (66%) overlapped with CpG islands, and the promoter region also showed overlap with the TSS200 and TSS1500 regions (25% and 24%, respectively) (Supplementary Figure S1). Analysis of individual CpG sites revealed few identifiable differentially methylated sites and roughly a balance of hypo- and hypermethylated sites (SmartSVA method, Table 6). In spite of the relatively low numbers of differentially methylated sites, we estimated that 9% (*n* = 27,276) of CpG sites were actually non-null. Out of all CpG sites represented in our analytical dataset (*n* = 313,161), 4–9% were estimated to be non-null CpG sites across individual gene regions (Table 6). Relatively fewer numbers of differentially methylated CpG sites were observed in analyses excluding SmartSVA (Supplementary Table S2).

2. Genes Associated with Differentially Methylated CpGs

Individual CpG sites that were differentially methylated were annotated to many of the genes identified as differential in the analysis of gene methylation. cg15613340 in *protocadherin beta 5* (*PCDHB5*) was significantly hypomethylated in vegans in the analysis of methylation of all sites and of the first exon, with a fold change of 0.81 (analyses with SmartSVA), and cg25026992 in *protocadherin beta 7* (*PCDHB7*) was similarly hypomethylated (fold change, 0.81) in analysis of the 3′ UTR region (analyses without SmartSVA), representing the most marked hypomethylation observed among CpG sites. The most marked hypermethylation was that of Cg07967210 in *SNF8 subunit of ESCRT-II* (*SNF8*), identified in the analysis of promoter methylation (fold change, 1.17) (Supplementary Tables S5–S8).

## 4. Discussion

DNA methylation is one mechanism that links lifestyle with genomic alterations. The goal of this study was to determine if habitual vegan and non-vegetarian dietary patterns differentially influenced DNA methylation. Using a permutation-based adapted Storey et al. [32] approach, and considering various genomic regions, we could detect a modest number of genes that differed significantly in their methylation status between vegans and non-vegetarians, noting that much larger proportions of apparently differentially methylated CpG sites and genes are in fact present, and should be detected with larger samples.

The majority of differentially methylated genes detected were hypomethylated in vegans, both when considering gene methylation overall, as well as in various gene regions. The greatest differences in gene methylation were found when considering the gene body. The region-specific differences in the number of detected differentially methylated genes are in part a reflection of the nonuniformity in the distribution of probes selected for the BeadChip array (i.e., a large number of CpG sites were present in the gene body). However, there were fewer observed and predicted non-null differentially methylated genes defined by the TSS1500 and CpG island regions, in spite of greater total numbers of genes with CpG sites in these regions, relative to the gene body. The gene body may therefore be the most susceptible to diet-induced alterations in methylation. Overall, we have estimated larger proportions of differentially methylated, non-null genes (6% of all genes) and CpG sites (9% of all CpG sites) to be detected with sufficiently large samples, using our adapted Storey approach. It should be noted that results from our permutation-based adapted Storey approach showed strong agreement with results from the simpler Benjamini–Hochberg approach for false discovery.

There is little other published evidence on the influence of plant-based dietary patterns on DNA methylation. Global methylation (LINE1) has been associated with consumption of fruits and vegetables or flavonoids in dietary intervention studies [41,42], as well as with energy restriction in overweight participants [43,44] although the latter has not been consistently observed [45]. It is unclear how a vegan diet impacted global (overall) methylation in the current study, given our array-based approach. However, of the significantly hypermethylated CpG sites, the majority were located in the intergenic region, which houses many transposable elements, including LINE1. Our findings show some consistency with those reported by Perfilyev et al., where high fat feeding (both saturated and polyunsaturated combined) was associated with hypermethylation of genes (>99%) [27]. These findings seem to complement our results as vegans who have lower fat consumption [9] and lower proportions of saturated fatty acids in adipose tissue [10], showed decreased gene methylation overall. However, the differential patterns of methylation between vegans and non-vegetarians in our study was not merely due to differences in fat, as our findings were only slightly attenuated when adjusted for total/saturated fat. Additionally, associations persisted when controlling for BMI, which could also be thought of as a surrogate of long-term energy intake and energy balance.

Many genes differentially methylated in the analysis of overall methylation of all genes have roles in RNA transport or ribosome assembly/regulation of translation (*RPL38*, *TUFM* [46,47]), as well as lysosome regulation (HPS4 [48]), protein degradation or ubiquitination (*DCAF15*, *PSMF1* [49,50]), cytokine or B cell receptor signaling (*CMTM7*, *IL36A* [51,52]), and metabolism (*STRA6*, *COX7A2L*, *GALNT12* [53,54,55,56]), among other pathways. Alterations in many of these processes impact protein synthesis and may have pathophysiological implications [57,58].

We have highlighted methylation differences in the promoter region particularly, as such alterations may have a greater impact on gene transcription. *METTL1* consistently showed hypomethylation in all relevant analyses—i.e., analyses of methylation of genes defined by CpGs in associated promoter regions, as well as of overall methylation of all genes, this being in analyses including or excluding SmartSVA variables, and after adjustment for false discovery. *METTL1* encodes a methyltransferase that promotes methylguanine methylation of RNA, including miRNAs such as let7, which results in processing of the miRNA [59]. Let-7 miRNA has roles in regulating cell growth and metabolism, and is also considered a tumor suppressor, showing downregulation in a number of cancers [60]. *METTL1* along with *NEIL2,* encoding an enzyme involved in DNA repair [61], were significantly hypomethylated in CpG islands within the promoter independent of SmartSVA variables. Hypermethylation of CpG islands within the promoter is associated with silencing of tumor suppressor genes [62,63,64,65]. Thus, hypomethylation and consequent increases in these genes might be associated with sustained expression of tumor suppressors, and thus cancer prevention.

Besides METTL1 and NEIL2, several of the differentially methylated genes identified by analysis of associated promoter methylation, or of all genes, have roles in cancer biology. For example, *LIN28B* regulates let-7 miRNA [66], *CMTM7* is potential tumor suppressor, possibly silenced by promoter methylation [49,67]), and *ARGHDIB*, a regulator of Rho family signaling, can be upregulated in tumors [68]. Interestingly, *GALNT12*, *DDT*, and *ARNT2* have been found to be over-represented in cancer, including colorectal cancer among others [69,70,71,72], with ARNT2 showing promoter hypermethylation in hepatoma cells [73]. We have found that vegans and other vegetarians in AHS-2 have lower risk of certain types of cancer and other chronic diseases [2,3,6]. Thus, DNA methylation alterations in genes with relevance to cancer could help explain some of the differences in cancer outcomes between dietary groups in the AHS-2 cohort. However, it is unclear if transcriptional changes are associated with any of the observed methylation alterations.

The observed trend towards hypomethylation of genes in vegans could be explained in part by inhibition of activity of DNMT enzymes by polyphenols and various secondary plant metabolites [14,74]. We have shown that vegetarians in the AHS-2 cohort have significantly increased consumption of plant-based foods including fruits, vegetables, legumes, nuts, and seeds [9], which are high in polyphenols. Furthermore, we recently demonstrated that vegetarians, particularly vegans, have significantly higher levels of bioactive compounds and phytochemicals-enterolactone, isoflavones, carotenoids, as well as total omega-3 fatty acids (attributable to alpha linolenic acid) in plasma, urine, and adipose tissue, many of which could theoretically modulate methylation [75]. Additionally, methyl donor compounds, folate, choline, methionine and cofactors (vitamin B), which are obtained from various foods, may influence both gene-specific and global DNA methylation, as demonstrated through observational and intervention studies; however, activity may be context, dose, or tissue dependent [13,30,76,77]. It is not clear if any of these methyl donor compounds differ between vegans and non-vegetarians in the current study. Thus, gene-specific and genome-wide alterations in methylation may be determined by a complex interplay of methyl donors, micronutrients, polyphenols, macronutrient composition, and inflammatory status, among other possible factors, including physical activity and stress. 

Methylation activity of CpGs within the same gene, and particularly the same region, may be coordinated. This correlation of CpG sites could partly explain why our analysis of gene methylation revealed greater differential alterations than analysis of individual CpG sites, although a much greater number of sites are estimated to be non-null and detectable with a larger sample. Findings from our study suggest that methylation alterations associated with dietary patterns (and likely other environmental exposures) may be characterized by coordinated methylation of CpG sites in a specific gene and within select regions of the gene. This approach of analyzing CpGs within select regions after annotation to their respective genes is, in general, similar to the region-centric approach taken by Bacalini et al. involving the grouping together of probes mapping to the same island or gene [78].

The effect size of methylation differences was low overall, with average regional methylation differences in the range of 2–4% for significant genes and 4–9% for significant CpG sites (at FDR < 0.05), though fold changes were much larger for select genes or CpG sites. Effect sizes of low magnitude (≤1–5%) have been previously reported in dietary studies. Besides the high fat feeding study by Perfilyev et al. [27], interventions examining alterations associated with a Mediterranean diet or diet enriched in flavonoids and isothiocyanates also found very subtle but significant changes (~1% methylation difference in LINE1) [41,44]. Smaller changes in methylation tend to be disregarded [79,80], but the physiological relevance of such small effects is unclear. It is possible that such changes over the long-term contribute to sustained, physiologically meaningful epigenetic differences. These “small” changes may translate into permanent changes in gene expression, and consequently phenotype, and be inherited by daughter cells [81]. The location of methylation alterations also holds much relevance, as methylation of one site can contribute to gene silencing if the methylated site blocks binding of a transcription factor [82,83].

This is the first study to our knowledge analyzing differences in DNA methylation between vegans and non-vegetarians. The AHS-2 cohort is unique in its relatively large number of vegans (~9% of cohort), thereby enabling such a study, although it should be noted that AHS-2 non-vegetarians tend to have lower meat consumption relative to the general population. The examination of habitual, long-term dietary patterns is a major strength, as epigenetic marks are more stable, reflecting long-term dietary conditions. We have shown that the majority of cohort members remain in the same diet group from one decade to the next [84]. Because of the habitual, a priori-classified dietary patterns, there was likely minimal measurement error in the classification of diet group. Additionally, our study was strengthened by leveraging multiple analytical approaches, comparing the SmartSVA method which adjusts for surrogate variables and thus unwanted variation, with linear regression models not including these surrogate variables but adjusting for other known technical variations.

Our study has some noteworthy limitations. Methylation alterations are best paralleled with gene expression data. In the absence of such data there are limitations in the interpretation of our findings. As mentioned, it is not clear how other dietary or lifestyle influences (exercise, methyl donors) may have altered results. Furthermore, we do not know how methylation alterations in leukocytes correlate with those in other tissues, although there is evidence that methylation patterns or alterations in blood may be reflective of patterns in other tissues [85,86,87]. Additionally, the two dietary groups examined were somewhat heterogeneous in terms of consumption of animal- and plant-based foods. For example, it is not clear how methylation patterns differ comparing individuals with very high consumption of red or processed meat with individuals following a diet comprised largely of fruits, vegetables, and whole plant foods.

## 5. Conclusions

In summary, modest specific differences in methylation of genes and CpG sites were detected, comparing vegans and non-vegetarians, with clear indication that many more such differences exist, but are yet to be specifically identified with larger studies. This study thus lays the foundation for the identification of transcriptional alterations and molecular functions associated with these diet-influenced methylation patterns.

## Figures and Tables

**Table 1:** Demographic characteristics of study population ^1^.

	Vegan	Non-Vegetarian	*p*
Participants (*n*)	62	142	
Sex (%)			0.57
Male	30 (48.4)	61 (43)	
Female	32 (51.6)	81 (57.0)	
Age (years)	67.3 (9.0)	65.9 (8.4)	0.28
BMI (kg/m^2^)	24.3 (4.6)	28 (5.1)	<0.001
Ethnicity (%)			0.50
Caucasian	46 (74.2)	97 (68.3)	
Black	16 (25.8)	45 (31.7)	

^1^ Values presented as mean (SD) or as *n* (%) where indicated.

**Table 2:** Estimated non-null and observed differentially methylated genes (false discovery rate < 0.05) summarized according to gene region or in relation to CpG islands comparing vegans with non-vegetarians ^1^.

Region ^2^	Total Genes		Estimated Non-Null		Significantly Hypermethylated		Significantly Hypomethylated	
	*n*	% of All Genes	*n* ^3^	% of Region-Specific Total	*n*	Fold Change ^4^	*n*	Fold Change ^4^
**All**	18,627	100	1081	5.8	4	1.02	14	0.97
**Genic/intergenic**								
TSS200	10,008	53.7	388	3.9	4	1.03	4	0.96
TSS1500	13,373	71.8	954	7.1	2	1.03	7	0.97
3′ UTR	1935	10.4	55	2.8	1	1.04	2	0.98
5′ UTR	7686	41.3	475	6.2	2	1.02	6	
Gene Body	12,072	64.8	1100	9.1	7	1.03	14	0.97
1st Exon	6262	33.6	405	6.5	1	1.07	9	0.97
Intergenic	8049	43.2	449	5.6	1	1.05	7	0.97
**Island-related**								
CpG Island	13,688	73.5	649	4.7	4	1.03	2	0.97
North Shelf	2562	13.8	180	7.0	2	1.03	2	0.97
North Shore	8315	44.6	424	5.1	1	1.03	7	0.96
South Shelf	2278	12.2	146	6.4	3	1.04	7	0.97
South Shore	7137	38.3	441	6.2	5	1.06	7	0.96
Open Sea	11,884	63.8	798	6.7	2	1.04	14	0.96
**Promoter**								
All	9131	49.0	459	5.0	2	1.05	9	0.98
CpG Island	7693	41.3	60	0.8	1	1.02	2	0.97

^1^ Number of actual differentially methylated genes determined using linear regression with SmartSVA followed by adapted Storey et al. [32] permutation approach to adjust for false discovery. ^2^ Individual CpGs may have been represented in more than one region when determining gene methylation of a given region. ^3^ Number of genes estimated to show non-null differences in methylation. ^4^ Fold change represents ratio of the mean methylation of vegans to that of non-vegetarians for differentially methylated (hypomethylated or hypermethylated) genes in a given region. This is averaged across significant genes.

**Table 3:** Numbers of genes expected to be detected with FDR < 0.05 as differentially methylated in vegans relative to non-vegetarians ^1^.

	Fold Increase in Sample Size—Hypomethylated Genes						Fold Increase in Sample Size—Hypermethylated Genes					
Region	1	1.5	2	3	4	5	1	1.5	2	3	4	5
All	14	28	42	66	79	86	4	29	103	266	377	456
Body	14	23	37	58	68	91	7	17	65	159	254	309
TSS1500	7	7	24	31	43	49	2	25	108	254	350	411
TSS200	4	4	4	12	19	19	4	9	44	141	203	254
3′ UTR	2	2	2	2	2	2	1	1	10	30	47	57
5′ UTR	6	6	12	17	26	28	2	15	47	122	172	226
1st Exon	9	10	23	33	37	43	1	10	45	96	142	169
Island	2	2	12	26	27	33	4	27	98	243	336	397
North Shore	7	7	12	23	24	28	1	8	47	144	222	260
South Shore	7	7	16	24	31	36	5	19	60	124	175	229
North Shelf	2	2	2	7	7	7	2	4	17	46	69	83
South Shelf	7	7	7	7	7	7	3	9	21	39	59	71
Promoter Associated	9	9	12	16	17	20	2	12	62	145	214	250
Promoter and CpG Island	2	2	2	5	10	13	1	8	45	112	161	201
Open Sea	14	20	28	44	58	61	2	11	61	144	218	256
Intergenic	7	8	19	30	40	51	1	7	40	101	154	185

^1^ Number of differentially methylated genes determined by adapted Storey et al. [32] based on plots of cumulative distributions of *p* values (derived from t statistics) from real and hypothetical null data.

**Table 4:** Differential methylation of genic/intergenic regions associated with individual genes at FDR < 0.05 (based on SmartSVA method) ^1^.

Gene ID	Gene Symbol	Description	Fold Change
**TSS200**			
341,350	OVCH1 (1)	ovochymase 1	0.93
8499	PPFIA2	PTPRF interacting protein alpha 2	0.96
51,099	ABHD5	abhydrolase domain containing 5, lysophosphatidic acid acyltransferase	0.98
51,205	ACP6	acid phosphatase 6, lysophosphatidic	0.98
9874	TLK1	tousled-like kinase 1	1.02
10,440	TIMM17A	translocase of inner mitochondrial membrane 17A	1.02
1132	CHRM4	cholinergic receptor, muscarinic 4	1.03
9570	GOSR2	lgi SNAP receptor complex member 2	1.03
**TSS1500**			
151,313	FAHD2B	fumarylacetoacetate hydrolase domain containing 2B	0.95
10,653	SPINT2	serine peptidase inhibitor, Kunitz type 2	0.96
100,302,640	LINC00882	Long Intergenic Non-Protein Coding RNA 882	0.96
84,838	ZNF496 (1)	zinc finger protein 496	0.96
4234	METTL1	methyltransferase-like 1	0.98
9054	NFS1	NFS1 cysteine desulfurase	0.98
1263	PLK3	polo-like kinase 3	0.98
8546	AP3B1	adaptor-related protein complex 3 subunit beta 1	1.02
11,267	SNF8	SNF8 subunit of ESCRT-II	1.04
**3′ UTR**			
79,960	JADE1	jade family PHD finger 1	0.97
667	DST	dystonin	0.98
54,820	NDE1	nudE neurodevelopment protein 1	1.04
**5′ UTR**			
252,969	NEIL2	nei-like DNA glycosylase 2	0.96
4147	MATN2	matrilin 2	0.97
5928	RBBP4	RB binding protein 4, chromatin remodeling factor	0.97
3792	KEL	Kell metallo-endopeptidase (Kell blood group)	0.97
9772	TMEM94	transmembrane protein 94	0.98
63,924	CIDEC	cell death-inducing DFFA-like effector c	0.99
51,465	UBE2J1	ubiquitin conjugating enzyme E2 J1	1.02
1374	CPT1A	carnitine palmitoyltransferase 1A	1.03
**Body**			
100,037,417	DDTL	D-dopachrome decarboxylase-like protein	0.92
154,007	SNRNP48	small nuclear ribonucleoprotein U11/U12 subunit 48	0.95
8120	AP3B2	adaptor-related protein complex 3 subunit beta 2	0.96
89,781	HPS4	HPS4, biogenesis of lysosomal organelles complex 3 subunit 2	0.96
2668	GDNF	glial cell line derived neurotrophic factor	0.96
56,666	PANX2	pannexin 2	0.97
91,010	FMNL3	formin-like 3	0.98
117,246	FTSJ3	FtsJ RNA 2’-O-methyltransferase 3	0.98
11,052	CPSF6	cleavage and polyadenylation-specific factor 6	0.98
149,297	FAM78B	family with sequence similarity 78 member B	0.98
399,671	HEATR4	HEAT repeat containing 4	0.98
285,987	DLX6-AS1	DLX6 antisense RNA 1	0.99
64,784	CRTC3	CREB regulated transcription coactivator 3	0.99
5361	PLXA1	plexin A1	0.99
**Body**			
9915	ARNT2	aryl hydrocarbon receptor nuclear translocator 2	1.02
5170	PDPK1	3-phosphoinositide dependent protein kinase 1	1.02
10,610	ST6GALNAC2	ST6 N-acetylgalactosaminide alpha-2,6-sialyltransferase 2	1.02
57,459	GATAD2B	GATA zinc finger domain containing 2B	1.03
3694	ITGB6	integrin subunit beta 6	1.04
58	ACTA1	actin alpha 1, skeletal muscle	1.05
9963	SLC23A1	solute carrier family 23 member 2	1.06
**First Exon**			
2201	FBN2	fibrillin 2	0.96
100,303,453	TSNAX-DISC1	Disrupted in schizophrenia 1 isoform 51	0.96
10,274	STAG1	stromal antigen 1	0.97
252,969	NEIL2	nei-like DNA glycosylase 2	0.97
3489	IGFBP6	insulin-like growth factor binding protein 6	0.97
5928	RBBP4	RB binding protein 4, chromatin remodeling factor	0.97
6727	SRP14	signal recognition particle 14	0.97
9167	COX7A2L (1)	cytochrome c oxidase subunit 7A2-like	0.97
1802	DPH2(1)	diphthamide biosynthesis 2	0.98
23,336	SYNM	Synemin	1.07
**All**			
100,037,417	DDTL	D-dopachrome tautomerase-like	0.95
284,680	SPATA46	spermatogenesis associated 46	0.96
100,113,403	LIN28B-AS1	LIN28B antisense RNA 1	0.96
64,220	STRA6	signaling receptor and transporter of retinol STRA6	0.97
4234	METTL1	methyltransferase-like 1	0.97
27,179	IL36A	interleukin 36 alpha	0.98
90,379	DCAF15	DDB1 and CUL4 associated factor 15	0.98
89,781	HPS4	HPS4, biogenesis of lysosomal organelles complex 3 subunit 2	0.98
136,371	ASB10	ankyrin repeat and SOCS box containing 10	0.98
397	ARHGDIB	Rho GDP dissociation inhibitor beta	0.98
6169	RPL38	ribosomal protein L38	0.98
55,222	LRRC20	leucine rich repeat containing 20	0.99
9491	PSMF1	proteasome inhibitor subunit 1	0.99
154,150	HDGFL1	HDGF-like 1	0.99
9915	ARNT2	aryl hydrocarbon receptor nuclear translocator 2	1.02
219,990	OOSP2	oocyte secreted protein 2	1.02
114,757	CYGB	cytoglobin	1.02
7284	TUFM	Tu translation elongation factor, mitochondrial	1.02
**Intergenic**			
136,371	ASB10	Ankyrin repeat and SOCS box protein 10	0.95
100,113,403	LIN28B-AS1	LIN28B antisense RNA 1	0.96
23,704	KCNE4	Potassium voltage-gated channel subfamily E member 4	0.97
27,319	BHLHE22	Class E basic helix-loop-helix protein 22	0.97
9935	MAFB	Transcription factor MafB	0.98
7100	TLR5	Toll-like receptor 5	0.98
154,150	HDGFL1	Hepatoma-derived growth factor-like protein 1	0.99
284,889	MIF-AS1	MIF antisense RNA	1.05

^1^ Number of differentially methylated CpG sites associated with each gene shown in brackets.

**Table 5:** Differential methylation of island-related and promoter regions associated with individual genes at FDR < 0.05 (based on SmartSVA method) ^1^.

Gene ID	Gene Symbol	Description	Fold Change
**Island**			
80,070	ADAMTS20	ADAM metallopeptidase with thrombospondin type 1 motif 20	0.95
101,409,261	OGFR-AS1	OGFR Antisense RNA 1	0.98
4609	MYC	MYC proto-oncogene	1.01
161,145	TMEM229B	transmembrane protein 229B	1.02
7284	TUFM	Tu translation elongation factor, mitochondrial	1.04
273	AMPH	amphiphysin	1.05
**North Shelf**			
4000	LMNA	lamin A/C	0.97
123,041	SLC24A4	solute carrier family 24 member 4	0.97
100,534,592	URGCP-MRPS24	URGCP-MRPS24 readthrough	1.02
116,844	LRG1	leucine rich alpha-2-glycoprotein 1	1.04
**North Shore**			
8022	LHX3	LIM homeobox protein 3	0.92
10,653	SPINT2	serine peptidase inhibitor, Kunitz type 2	0.95
6169	RPL38	ribosomal protein L38	0.96
23,200	ATP11B	Probable phospholipid-transporting ATPase IF	0.96
7707	ZNF148	Zinc finger protein 148	0.97
26,156	RSL1D1	ribosomal L1 domain containing 1	0.97
9379	NRXN2	Neurexin-2	0.98
9480	ONECUT2	one cut homeobox 2	1.03
**South Shelf**			
55,608	ANKRD10	ankyrin repeat domain 10	0.95
26,693	OR2V1	olfactory receptor family 2 subfamily V member 1	0.96
2180	ACSL1	acyl-CoA synthetase long chain family member 1	0.96
399,829	LINC01168	long intergenic non-protein coding RNA 1168	0.97
2774	GNAL	G protein subunit alpha L	0.97
64,319	FBRS	fibrosin	0.97
84,286	TMEM175	transmembrane protein 175	0.97
140,706	CCM2L	cerebral cavernous malformation 2-like	1.04
1112	FOXN3	forkhead box N3	1.04
10,410	IFITM3	interferon-induced transmembrane protein 3	1.04
**South Shore**			
41	ASIC1	acid-sensing (proton-gated) ion channel 1	0.94
151,313	FAHD2B	fumarylacetoacetate hydrolase domain containing 2B	0.95
84,838	ZNF496 (2)	zinc finger protein 496	0.95
116,988	AGAP3	ArfGAP with GTPase domain, ankyrin repeat and PH domain 3	0.95
9735	KNTC1	kinetochore associated 1	0.96
29,993	PACSIN1	protein kinase C and casein kinase substrate in neurons 1	0.96
85,395	FAM207A	family with sequence similarity 207 member A	0.97
57,186	RALGAPA2	Ral GTPase activating protein catalytic subunit alpha 2	1.03
55,243	KIRREL1	kirre-like nephrin family adhesion molecule 1	1.03
11,267	SNF8	SNF8 subunit of ESCRT-II	1.04
375,061	FAM89A	family with sequence similarity 89, member A	1.06
57,495	NWD2	NACHT and WD repeat domain containing 2	1.12
**Open Sea**			
25,774	GSTTP1	glutathione S-transferase theta 4	0.72
402,381	SOHLH1	spermatogenesis and oogenesis specific basic helix-loop-helix 1	0.96
3034	HAL	histidine ammonia-lyase	0.96
64,220	STRA6	signaling receptor and transporter of retinol STRA6	0.97
134,510	UBLCP1	ubiquitin-like domain containing CTD phosphatase 1	0.97
1188	CLCNKB	Chloride channel protein ClC-Kb	0.97
1610	DAO	D-amino-acid oxidase	0.98
397	ARHGDIB	Rho GDP-dissociation inhibitor 2	0.98
27,179	IL36A	interleukin 36 alpha	0.98
54,989	ZNF770	zinc finger protein 770	0.98
83,394	PITPNM3	PITPNM family member 3	0.98
79,981	FRMD1	FERM domain containing 1	0.98
55,137	FIGN	fidgetin	0.99
114,879	OSBPL5	oxysterol binding protein-like 5	0.99
219,990	OOSP2	oocyte secreted protein 2	1.02
100,130,673	LOC100130673	phosphoribosyl pyrophosphate synthetase 2 pseudogene	1.06
**Promoter**			
55,640	FLVCR2	FLVCR heme transporter 2	0.95
79,695	GALNT12	polypeptide N-acetylgalactosaminyltransferase 12	0.96
6169	RPL38	ribosomal protein L38	0.97
4234	METTL1	methyltransferase-like 1	0.97
112,616	CMTM7	CKLF-like MARVEL transmembrane domain containing 7	0.98
10,073	SNUPN	snurportin 1	0.98
80,321	CEP70	centrosomal protein 70	0.98
9167	COX7A2L	cytochrome c oxidase subunit 7A2-like	0.99
100,288,142	NBPF20	NBPF member 20	0.99
8546	AP3B1	adaptor-related protein complex 3, beta 1 subunit	1.01
100,506,334	LINC00649 (1)	long intergenic non-protein coding RNA 649	1.08
**Islands in Promoter**			
4234	METTL1	methyltransferase-like 1	0.97
252,969	NEIL2	nei-like DNA glycosylase 2	0.97
200,312	RNF215	ring finger protein 215	1.02

^1^ Number of differentially methylated CpG sites associated with each gene shown in parentheses.

**Table 6:** Estimated non-null and observed differentially methylated CpG sites (FDR < 0.05) summarized according to genic/intergenic region or in relation to CpG islands comparing vegans with non-vegetarians ^1^.

Region ^2^	Total Genes		Estimated Non-Null		Significantly Hypermethylated		Significantly Hypomethylated	
	*n*	% of All CpG Sites	*n* ^3^	% of Region-Specific Total	*n*	Fold Change ^4^	*n*	Fold Change ^4^
**All**	313,161	100	27,276	8.7	6	1.06	8	0.92
**Genic/intergenic**								
TSS200	40,816	13.0	2355	5.8	5	1.05	3	0.91
TSS1500	57,450	18.3	3950	6.9	5	1.06	8	0.93
3′ UTR	12,840	4.1	633	4.9	3	1.04	6	0.93
5′ UTR	43,313	13.8	3270	7.6	4	1.08	3	0.95
Gene Body	114,095	36.4	9835	8.6	7	1.08	6	0.95
1st Exon	25,662	8.2	2082	8.1	1	1.04	7	0.92
Intergenic	72,524	23.2	5798	8.0	9	1.06	3	0.92
**Island-related**								
CpG Island	99,350	31.7	7883	7.9	4	1.07	4	0.92
North Shelf	15,106	4.8	923	6.1	7	1.05	6	0.93
North Shore	43,549	13.9	3542	8.1	6	1.06	3	0.94
South Shelf	13,564	4.3	974	7.2	9	1.06	3	0.93
South Shore	33,905	10.8	2652	7.8	5	1.08	7	0.93
Open Sea	107,687	34.4	8266	7.7	5	1.06	5	0.93
**Promoter**								
All	62,398	19.9	2308	3.7	6	1.08	3	0.95
CpG Island	39,230	12.5	1713	4.4	1	1.07	3	0.96

^1^ Number of differentially methylated CpG sites determined using linear regression with SmartSVA, followed by adapted Storey et al. [32] approach to adjust for false discovery. ^2^ Some CpG sites represented in more than one gene region. ^3^ Number of CpG sites estimated to show non-null differences in methylation. ^4^ Fold change represents ratio of the mean methylation of vegans to that of non-vegetarians for differentially methylated (hypomethylated or hypermethylated) sites for a given region. This is averaged across significant sites.

**Figure A2:**
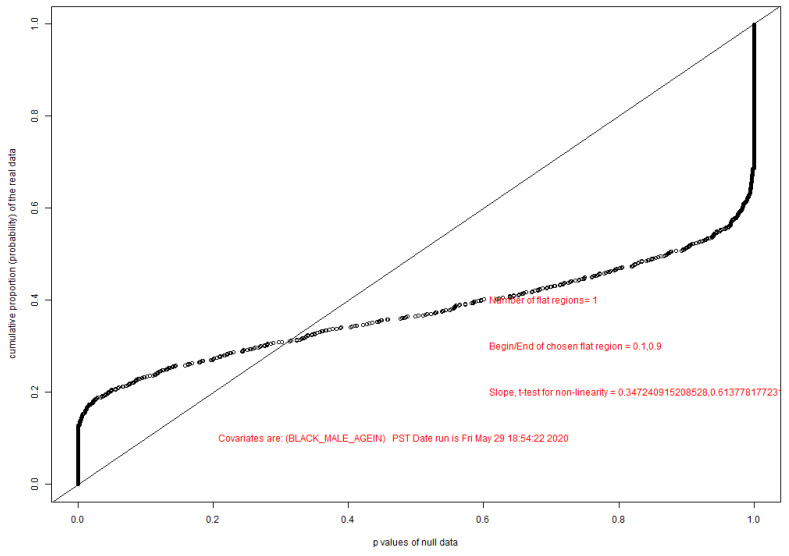
Cumulative *p* plots (real and hypothetical null data) for a metabolomics panel comparing non-vegetarians to vegans.

## Supplementary Materials

The following are available online at https://www.mdpi.com/2072-6643/12/12/3697/s1. Figure S1: Overlap of promoter with gene- or island-related regions, Table S1: Estimated non-null and observed differentially methylated genes (FDR < 0.05) summarized according to genic/intergenic region or in relation to CpG islands in vegans relative to non-vegetarians (without SmartSVA method), Table S2: Estimated non-null and observed differentially methylated CpG sites (at FDR < 0.05) summarized according to genic/intergenic region or in relation to CpG islands in vegans relative to non-vegetarians (without SmartSVA), Table S3: Genes differentially methylated at FDR < 0.05 according to genic/intergenic region, Table S4: Genes differentially methylated at FDR < 0.05 in island-related and promoter regions, Table S5: CpGs differentially methylated at FDR < 0.05 according to genic/intergenic region (based on SmartSVA method), Table S6: CpGs differentially methylated at FDR < 0.05 in island-related and promoter regions (based on SmartSVA method), Table S7: CpGs differentially methylated at FDR < 0.05 according to genic/intergenic region, Table S8: CpGs differentially methylated at FDR < 0.05 in island-related and promoter regions.

## Appendix A

### A Method of Power Calculations, Estimating Ability to Detect Differentially Methylated Sites with a Given False Discovery Rate

First, in what follows, *p* values are defined as the cumulative probabilities of the chosen test statistics when the null hypothesis is satisfied, thus ranging from 0 to 1.0. As noted by Storey et al. [32], a plot of the density of *p* values for a central t distribution (or other distributions) is a uniform plot, when the data conform to the null (the hypothesis that the *p* value is defined to represent). Then, it is assumed that the observed data to be evaluated (consisting of a large number, say R, independent *t* statistics), represent a mix from two types of CpG (or gene) populations. Namely, these are a population of true null data, and a second population where the true distribution for each *t* statistic is non-null (non-central), but not otherwise specified.

In that situation the density plot of *p* values (estimated, say, from a central t distribution) should devolve into a central uniform portion, at a density less than 1.0, say R_0_/R, and tails at either end that slope upward from the uniform region represent an excess of *p* values in regions more sparsely populated under the null. The total area under the curve must, as usual, equal 1.0, by the definition of density. One can compare this to a plot that would be expected if all values came from a null distribution, which would be a uniform plot at a value of 1.0. It is easy to show that the value R_0_/R represents the proportion of the t values in the real data that come from a null distribution. Then R—R_0_ is the estimated number of t statistics that come from non-null distributions.

To gain some extra stability, we have simply translated this same rationale to cumulative distributions of *p* values from both observed data and hypothetical null data. In the null situation the cumulative distribution is a diagonal line from lower left to upper right. In the observed data, the central uniform density cumulates to a straight line that can be shown to have slope R_0_/R. Relative excess densities at small *p* values cumulate to push the left end of the curve higher than the diagonal with slopes that exceed 1.0, thus flattening the straight line null portion that begins at the end of this elevated segment. Unless there are no non-null data at the right-hand higher-valued end, the central straight portion necessarily crosses the diagonal. Then later, at the end of the null straight portion, again develops slopes greater than 1.0, representing non-null high-value P statistics, finally meeting the diagonal again when *p* = 1.0. In these plots the slope of the straight line portion estimates the proportion of underlying null distributions, and of course again (R—R_0_)/R is the proportion that are non-null.

An example from the DNA methylation data (average of CpG sites in gene body regions) described in this paper is shown as Figure A1. Here, the deviation in slope of the central linear region from the diagonal is relatively small, but important, and in part due to large numbers, is not due to chance (*p* < 2 × 10^−16^). The size of the population studies, and the relatively small fold change values (that translate to smaller non-centrality parameters for the non-null t distributions) account for the modest deviation from the diagonal in the figure. Increasing *n* (the number of subjects) will change this (see Table 4). An example from a metabolomics dataset—also comparing non-vegetarians to vegans—includes t distributions with much greater non-centrality parameters and is shown below for illustrative purposes (Figure A2).

This situation can be used to estimate power, and how, in a particular dataset, this depends on *n*. Power is here expressed as the false discovery rate (FDR) associated with sample size *n*2, where *n*1 is the size of the observed data, and where critical *t* statistic values are nominated for exploratory purposes. This is dependent on the observation that as *n* increases, the distribution of *t* statistics from null sites does not change. The reason for this is that the numerator of each *t* value is (mean(non-vegetarians)—mean(vegans)) for that site or gene, and its expectation by definition under the null, is zero. Thus, the underlying random errors in these means will shrink as *n* increases according to the inverse square root law, decreasing the range of the random differences between the two means (the numerator). The denominator that measures the standard error of this difference between means shrinks by an exactly equivalent amount; however, the ratio *t* statistic remains unchanged, with other things being equal.

However, the situation for non-null t values is different—the numerators, again by definition, have a non-zero expected value (that differs from methylation site to methylation site). As *n* increases, this expected value does not change, but the denominator (Standard Error of the difference) shrinks as usual, driving the non-null *t* statistics to ever more extreme values. This has the most helpful consequence of drawing the non-null results ever more toward the tails of the observed distribution as numbers increase. So, for any nominated critical t value, the tails will contain the usual number of randomly extreme *t* statistics from null sites, but an increasing number from non-null sites with increasing numbers of subjects, thus improving the FDR.

Specifically, it can be shown that, for the left-hand tail,

FDR(n2) = $\frac{\mathrm{FDR}\left(n1\right).R.\mathrm{cp}\left(\mathrm{Tcl}\right)}{\left\{R.\mathrm{cp}\left(\mathrm{Tcl}.\surd \left(n1/n2\right)\right)\;–\;R_{0}.\left[p\left(\mathrm{Tcl}.\surd \left(n1/n2\right)\right)\;-\;p\left(\mathrm{Tcl}\right)\right]\right\}}$, where Tcl is the nominated critical *t* value for the left-hand tail; p(Tcl) refers to the cumulative probability under the null corresponding to this *t* value; cp(Tcl) refers to the cumulative probability of this Tcl value in the observed data.

For the right-hand tail where Tcu is the critical value for significance in the upper tail, the expression is

$$\mathrm{FDR}(n2)\;=\;\frac{\mathrm{FDR}\left(n1\right).R.\left(1-\mathrm{cp}\left(\mathrm{Tcu}\right)\right)\;}{\left\{R.\left(1-\mathrm{cp}\left(\mathrm{Tcu}.\surd \left(n1/n2\right)\right)\right)\;–\;R_{0}.\left[p\left(\mathrm{Tcu}\right)\;-\;p\left(\mathrm{Tcu}.\surd \left(n1/n2\right)\right)\right]\right\}}.$$
