# Supplementary material for: DNA Methylation Profiles of Vegans and Non-Vegetarians in the Adventist Health Study-2 Cohort
Source: Nutrients. 2020 Nov 30;12(12):3697. doi: 10.3390/nu12123697 (PMC7761449; doi:10.3390/nu12123697)
Supplement: Supplementary file 1 [file nutrients-12-03697-s001.pdf]

Supplementary Table S1. Estimated non-null and observed differentially methylated genes (FDR < 0.05) summarized according to genic/intergenic region or in relation to CpG islands in vegans relative to non-vegetarians (without SmartSVA method) [1]

Region[2]	Total Genes		Estimated Non-null		Significantly Hypermethylated		Significantly Hypomethylated	
	n	% of all genes	n[3]	% of region-specific total	n	Fold Change[4]	n	Fold Change[4]
**All**	18627	100	1	0	0	NA	5	0.97
**Genic/intergenic**								
TSS200	10008	54	0	0	0	NA	4	0.97
TSS1500	13373	72	1	0	0	NA	3	0.95
3' UTR	1935	10	152	7.8	0	NA	0	NA
5' UTR	7686	41	0	0	0	NA	0	NA
Gene Body	12072	65	1	0	0	NA	5	0.95
1st Exon	6262	34	1	0	0	NA	3	0.95
Intergenic	8049	43	1,642	20.4	0	NA	3	0.91
**Island-related**								
CpG Island	13688	73	0	0	0	NA	1	0.97
North Shelf	2562	14	879	34.3	0	NA	2	0.94
North Shore	8315	45	0	0	0	NA	2	0.94
South Shelf	2278	12	2	0.1	1	1.04	1	0.95
South Shore	7137	38	1	0	0	NA	2	0.93
Open Sea	11884	64	459	3.9	0	NA	1	0.74
**Promoter**								
All	9131	49	741	8.1	0	NA	6	0.97
CpG Island	7693	41	993	12.9	0	NA	2	0.97

[1] Number of actual differentially methylated genes determined using multivariate linear regression followed by adapted Storey et al. [1] permutation approach to adjust for false discovery. [2] Individual CpGs may have been represented in more than one region when determining gene methylation of a given region. [3] Number of genes estimated to show non-null differences in methylation. [4] Fold change represents ratio of the mean methylation of vegans to that of non-vegetarians for differentially methylated genes.

Supplementary Table S2. Estimated non-null and observed differentially methylated CpG sites (at FDR < 0.05) summarized according to genic/intergenic region or in relation to CpG islands in vegans relative to non-vegetarians (without SmartSVA)[1]

Region[2]	Total Genes		Estimated Non-null		Significantly Hypermethylated		Significantly Hypomethylated	
	n	% of all CpG sites	n[3]	% of region-specific total	n	Fold Change[4]	n	Fold Change[4]
**All**	313161		0	0	4	1.09	1	0.95
**Genic/intergenic**								
TSS200	40816	13	0	0	0	NA	2	0.92
TSS1500	57450	18	0	0	4	1.06	2	0.93
3' UTR	12840	4	0	0	2	1.06	5	0.9
5' UTR	43313	14	0	0	3	1.1	1	0.9
Gene Body	114095	36	0	0	5	1.07	0	NA
1st Exon	25662	8	0	0	1	1.06	5	0.88
Intergenic	72524	23	0	0	6	1.07	0	NA
**Island-related**								
CpG Island	99350	32	5951	6	4	1.11	1	0.81
North Shelf	15106	5	4628	31	5	1.07	2	0.86
North Shore	43549	14	1	0	2	1.06	2	0.95
South Shelf	13564	4	4044	30	2	1.06	3	0.93
South Shore	33905	11	0	0	3	1.06	3	0.89
Open Sea	107687	34	38272	36	4	1.09	0	NA
**Promoter**								
Promoter	62398	20	4210	7	0	NA	1	0.95
Island in Promoter	39230	13	0	0	2	1.12	6	0.93

[1] Number of differentially methylated CpG sites determined using multivariate linear regression, followed by adapted Storey et al. [1] approach to adjust for false discovery.
[2] Some CpG sites represented in more than one gene region. [3] Number of CpG sites estimated to show non-null differences in methylation. [4] Fold change represents ratio of the mean methylation of vegans to that of non-vegetarians for differentially methylated genes.

Supplementary Table S3. Genes differentially methylated at FDR < 0.05 according to genic/intergenic region1.

Gene ID	Gene Symbol	Description	Fold Change
**TSS200**			
57035	RSRP1	arginine and serine rich protein 1	0.96
3592	IL12A	interleukin 12A	0.97
7161	TP73	tumor protein p73	0.97
9491	PSMF1	proteasome inhibitor subunit 1	0.98
**TSS1500**			
26281	FGF20	fibroblast growth factor 20	0.91
924	CD7	CD7 molecule	0.97
4234	METTL1	methyltransferase like 1	0.98
**Body**			
100037417	DDTL	D-dopachrome tautomerase like	0.93
8022	LHX3	LIM homeobox 3	0.94
128954	GAB4	GRB2 associated binding protein family member 4	0.94
11052	CPSF6	cleavage and polyadenylation specific factor 6	0.97
117246	FTSJ3	FtsJ RNA 2'-O-methyltransferase 3	0.98
**All**			
100113403	LIN28B-AS1	LIN28B antisense RNA 1	0.95
8022	LHX3	LIM homeobox 3	0.96
55640	FLVCR2	FLVCR heme transporter 2	0.96
4234	METTL1	methyltransferase like 1	0.98
9491	PSMF1	proteasome inhibitor subunit 1	0.98
**Intergenic**			
2928	GSC2	1,3-beta-glucan synthase GSC2	0.88
403315	FAM92A1P2	CIBAR1 pseudogene 2	0.90
100113403	LIN28B-AS1	LIN28B antisense RNA 1	0.95

[1]Linear regression model does not include surrogate variables.

Supplementary Table S4. Genes differentially methylated at FDR < 0.05 in island-related and promoter regions[1].

Gene ID	Gene Symbol	Description	Fold Change
**Island**			
27074	LAMP3	lysosomal-associated membrane protein 3	0.97
**North Shelf**			
253769	WDR27	WD repeat domain 27	0.94
133957	CCDC127	coiled-coil domain containing 127	0.95
**North Shore**			
8022	LHX3	LIM homeobox 3	0.92
6169	RPL38	ribosomal protein L38	0.97
**South Shelf**			
3169	FOXA1	forkhead box A1	0.95
6624	FSCN1	fascin actin-bundling protein 1	1.04
**South Shore**			
55640	FLVCR2	FLVCR heme transporter 2	0.91
6794	STK11	serine/threonine kinase 11	0.95

**Open Sea**			
25774	GSTTP1	glutathione S-transferase theta 4	0.74
**Promoter**			
55640	FLVCR2	FLVCR heme transporter 2	0.94
4234	METTL1	methyltransferase like 1	0.97
6169	RPL38	ribosomal protein L38	0.97
112616	CMTM7	CKLF like MARVEL transmembrane domain containing 7	0.97
10073	SNUPN	Snurportin 1	0.98
55207	ARL8B	ADP Ribosylation Factor Like GTPase 8B	0.98
**Islands in Promoter**			
4234	METTL1	methyltransferase like 1	0.97
252969	NEIL2	nei like DNA glycosylase 2	0.97

[1]Linear regression model does not include surrogate variables.

Supplementary Table S5. CpGs differentially methylated at FDR < 0.05 according to genic/intergenic region (based on SmartSVA method).

Site label	Gene ID	Gene Symbol	Description	Fold Change
**TSS200**				
cg07854132	341350	OVCH1	ovochymase 1	0.86
cg04690395	130497	OSR1	odd-skipped related transcription factor 1	0.92
cg09232937	79192	IRX1	iroquois homeobox 1	0.95
cg16862361	7498	XDH	xanthine dehydrogenase	1.04
cg01815090	4151	MB	myoglobin	1.05
cg17015937	200197	TMEM51-AS1	TMEM51 antisense RNA 1	1.05
cg07280097	2815	GP9	glycoprotein IX platelet	1.06
cg18785300	3106	HLA-B	major histocompatibility complex, class I, B	1.07
**TSS1500**				
cg02221302	100506994	PTPRG-AS1	PTPRG antisense RNA 1	0.87
cg19725553	644	BLVRA	biliverdin reductase A	0.90
cg22892237	400684	LOC400684	uncharacterized LOC400684	0.93
cg24404823	84838	ZNF496	zinc finger protein 496	0.94
cg06052471	51524	TMEM138	transmembrane protein 138	0.95
cg00776160	723790	HIST2H2AA4	H2A clustered histone 19	0.96
cg05930166	64853	AIDA	axin interactor, dorsalization associated	0.96
cg21169617	25822	DNAJB5	DnaJ heat shock protein family	0.96
cg21209815	79095	C9orf16	chromosome 9 open reading frame 16	1.04
cg23228694	22839	DLGAP4	DLG associated protein 4	1.05
cg17015937	200197	TMEM51-AS1	TMEM51 antisense RNA 1	1.06
cg17372806	10313	RTN3	reticulon 3	1.07
cg02103875	83450	DRC3	dynein regulatory complex subunit 3	1.07
**3' UTR**				
cg01644592	4340	MOG	myelin oligodendrocyte glycoprotein	0.90
cg21256200	391322	LOC391322	D-dopachrome tautomerase-like	0.93
cg15173079	7280	TUBB2A	tubulin beta 2A class IIa	0.93
cg19054685	57176	VARS2	valyl-tRNA synthetase 2, mitochondrial	0.94
cg00452252	6340	SCNN1G	sodium channel epithelial 1 subunit gamma	0.94
cg09152853	81789	TIGD6	tigger transposable element derived 6	0.96
cg11234767	595	CCND1	cyclin D1	1.02
cg18811916	55161	TMEM33	transmembrane protein 33	1.03
cg26915799	55705	IPO9	importin 9	1.07
**5' UTR**				
cg02621907	84250	SLF1	SMC5-SMC6 complex localization factor 1	0.95
cg06052471	51524	TMEM138	transmembrane protein 138	0.95
cg07968927	283489	CHAMP1	chromosome alignment maintaining phosphoprotein 1	0.96
cg04275030	221178	SPATA13	spermatogenesis associated 13	1.05
cg06320606	79993	ELOVL7	ELOVL fatty acid elongase 7	1.06
cg02103875	83450	DRC3	dynein regulatory complex subunit 3	1.07
cg02430797	55753	OGDHL	oxoglutarate dehydrogenase L	1.15

Supplementary Table S6. CpGs differentially methylated at FDR < 0.05 in island-related and promoter regions (based on SmartSVA method).

Site label	Gene ID	Gene Symbol	Description	Fold Change
**North Shelf**				
cg04442426	2668	GDNF	glial cell derived neurotrophic factor	0.89
cg07367222	63976	PRDM16	PR domain containing 16	0.92
cg25727554	3833	KIFC1	kinesin family member C1	0.94
cg17386710	26053	AUTS2	activator of transcription and developmental regulator AUTS2	0.95
cg18098567	389033	PLAC9P1	placenta associated 9 pseudogene 1	0.95
cg24315049	85452	CFAP74	cilia and flagella associated protein 74	0.95
cg19272986	65989	DLK2	delta like non-canonical Notch ligand 2	1.03
cg08121523	25885	POLR1A	RNA polymerase I subunit A	1.04
cg01558281	476	ATP1A1	ATPase Na+/K+ transporting subunit alpha 1	1.04
cg11944784	53346	TM6SF1	transmembrane 6 superfamily member 1	1.05
cg22746351	9441	MED26	mediator complex subunit 26	1.05
cg05838751	64788	LMF1	lipase maturation factor 1	1.06
cg18034859	85366	MYLK2	myosin light chain kinase 2	1.06
**North Shore**				
cg00462362	54869	EPS8L1	EPS8 like 1	0.90
cg05930166	64853	AIDA	axin interactor, dorsalization associated	0.96
cg21169617	25822	DNAJB5	DnaJ heat shock protein family (Hsp40) member B5	0.96
cg06116255	6656	SOX1	SRY-box transcription factor 1	1.04
cg05794042	201501	ZBTB7C	zinc finger and BTB domain containing 7C	1.05
cg17015937	200197	TMEM51-AS1	TMEM51 antisense RNA 1	1.06
cg07994487	9742	IFT140	intraflagellar transport 140	1.07
cg17372806	10313	RTN3	reticulon 3	1.07
cg02243714	57338	JPH3	junctophilin 3	1.08
**South Shelf**				
cg07308232	84310	C7orf50	chromosome 7 open reading frame 50	0.88
cg06204629	257194	NEGR1	neuronal growth regulator 1	0.94
cg08489478	118813	ZFYVE27	zinc finger FYVE-type containing 27	0.95
cg11937362	80856	LNPK	lunapark, ER junction formation factor	1.03
cg19780614	23303	KIF13B	kinesin family member 13B	1.03
cg21696636	64359	NXN	nucleoredoxin	1.04
cg16336066	79789	CLMN	calmin	1.04
cg10736454	199675	MCEMP1	mast cell expressed membrane protein 1	1.05
cg15412087	220323	OAF	out at first homolog	1.05
cg00599463	140706	CCM2L	CCM2 like scaffold protein	1.07
cg08229058	479	ATP12A	ATPase H+/K+ transporting non-gastric alpha2 subunit	1.08
cg11496466	6433	SFSWAP	splicing factor SWAP	1.12
**South Shore**				
cg24846343	100037417	DDTL	D-dopachrome decarboxylase-like protein	0.87
cg13911707	84838	ZNF496	zinc finger protein 496	0.92
cg21256200	391322	LOC391322	D-dopachrome tautomerase-like	0.93
cg24404823	84838	ZNF496	zinc finger protein 496	0.94
cg06052471	51524	TMEM138	transmembrane protein 138	0.95
cg00776160	723790	HIST2H2AA4	H2A clustered histone 19	0.96
cg02569596	54090	LINC00111	long intergenic non-protein coding RNA 111	0.97
cg00899907	57819	LSM2	LSM2 homolog, U6 small nuclear RNA and mRNA degradation associated	1.06
cg13893634	84687	PPP1R9B	protein phosphatase 1 regulatory subunit 9B	1.06
cg03721195	2676	GFRA3	GDNF family receptor alpha 3	1.07
cg02103875	83450	DRC3	dynein regulatory complex subunit 3	1.08
cg26333393	119587	CPXM2	carboxypeptidase X, M14 family member 2	1.12
**Open Sea**				
cg07854132	341350	OVCH1	ovochymase 1	0.85
cg11413576	9760	TOX	thymocyte selection associated high mobility group box	0.92
cg15215077	4121	MAN1A1	mannosidase alpha class 1A member 1	0.95
cg09152853	81789	TIGD6	tigger transposable element derived 6	0.96
cg03335128	2782	GNB1	G protein subunit beta 1	0.96
cg20639263	4860	PNP	purine nucleoside phosphorylase	1.05
cg07280097	2815	GP9	glycoprotein IX platelet	1.05
cg23229110	4921	DDR2	discoidin domain receptor tyrosine kinase 2	1.06
cg14479306	158135	TTLL11	tubulin tyrosine ligase like 11	1.06
cg17423032	23037	PDZD2	PDZ domain containing 2	1.06
cg25935697	132141	IQCF1	IQ motif containing F1	1.10

**Promoter**				
cg09048186	165324	UBXN2A	UBX domain protein 2A	0.94
cg06052471	51524	TMEM138	transmembrane protein 138	0.96
cg21169617	25822	DNAJB5	DnaJ heat shock protein family (Hsp40) member B5	0.96
cg20639263	4860	PNP	purine nucleoside phosphorylase	1.05
cg17883742	9422	ZNF264	zinc finger protein 264	1.05
cg17372806	10313	RTN3	reticulon 3	1.07
cg18785300	3106	HLA-B	major histocompatibility complex, class I, B	1.07
cg08309687	100506334	LINC00649	long intergenic non-protein coding RNA 649	1.09
cg07967210	11267	SNF8	SNF8 subunit of ESCRT-II	1.17
**Islands in Promoter**				
cg07968927	283489	CHAMP1	chromosome alignment maintaining phosphoprotein 1	0.96
cg13897296	23528	ZNF281	zinc finger protein 281	0.96
cg24336123	9054	NFS1	NFS1 cysteine desulfurase	0.98
cg18785300	3106	HLA-B	major histocompatibility complex, class I, B	1.07

Supplementary Table S7. CpGs differentially methylated at FDR < 0.05 according to genic/intergenic region[1.]

	Gene ID	Gene Symbol	Description	Fold Change
**TSS200**				
cg16720650	100507266	STX18-AS1	STX18 antisense RNA 1	0.91
cg17452713	80818	ZNF436	zinc finger protein 436	0.94
**TSS1500**				
cg07190921	3483	IGFALS	insulin like growth factor binding protein acid labile subunit	0.91
cg21169617	25822	DNAJB5	DnaJ heat shock protein family (Hsp40) member B5	0.95
cg01618535	400866	LINC00114	long intergenic non-protein coding RNA 114	1.04
cg12562418	5919	RARRES2	retinoic acid receptor responder 2	1.04
cg15841865	285965	EPHA1-AS1	EPHA1 antisense RNA	1.07
cg02103875	83450	DRC3	dynein regulatory complex subunit 3	1.09
**3' UTR**				
cg25026992	56129	PCDHB7	protocadherin beta 7	0.81
cg01644592	4340	MOG	myelin oligodendrocyte glycoprotein	0.89
cg05938001	1745	DLX1	distal-less homeobox 1	0.92
cg19054685	57176	VARS2	valyl-tRNA synthetase 1	0.93
cg08296969	54921	CHTF8	chromosome transmission fidelity factor 8	0.94
cg09072576	54584	GNB1L	G protein subunit beta 1 like	1.04
cg21434327	2309	FOXO3	forkhead box O3	1.08
**5' UTR**				
cg13899780	22941	SHANK2	SH3 and multiple ankyrin repeat domains 2	0.90
cg15381410	4232	MEST	mesoderm specific transcript	1.04
cg02103875	83450	DRC3	dynein regulatory complex subunit 3	1.09
cg02430797	55753	OGDHL	oxoglutarate dehydrogenase L	1.17

[1] Linear regression model does not include surrogate variables.

Supplementary Table 7, cont. CpGs differentially methylated at FDR < 0.05 according to genic/intergenic region[1.]

Site label	Gene ID	Gene Symbol	Description	Fold Change
**Body**				
cg15381410	4232	MEST	mesoderm specific transcript	1.04
cg23229110	4921	DDR2	discoidin domain receptor tyrosine kinase 2	1.07
cg07114544	9423	NTN1	netrin 1	1.07
cg08130814	79648	MCPH1	microcephalin 1	1.09
cg00699321	5799	PTPRN2	protein tyrosine phosphatase receptor type N2	1.09
**First Exon**				
cg25026992	56129	PCDHB7	protocadherin beta 7	0.81
cg15613340	26167	PCDHB5	protocadherin beta 5	0.81
cg26752888	252969	NEIL2	nei like DNA glycosylase 2	0.89
cg05201673	4722	NDUFS3	NADH:ubiquinone oxidoreductase core subunit S3	0.93
cg15811902	10073	SNUPN	snurportin 1	0.94

cg18750756	201305	SPNS3	sphingolipid transporter 3	1.06
**All**				
cg21169617	25822	DNAJB5	DnaJ heat shock protein family (Hsp40) member B5	0.95
cg09171355	81848	SPRY4	sprouty RTK signaling antagonist 4	1.06
cg23229110	4921	DDR2	discoidin domain receptor tyrosine kinase 2	1.07
cg25935697	132141	IQCF1	IQ motif containing F1	1.11
cg19769989	51058	ZNF691	zinc finger protein 691	1.11
**Intergenic**				
cg04649441	10910	SUGT1	SGT1 homolog, MIS12 kinetochore complex assembly cochaperone	1.05
cg06116255	6656	SOX1	SRY-box transcription factor 1	1.05
cg16336066	79789	CLMN	calmin	1.05
cg09171355	81848	SPRY4	sprouty RTK signaling antagonist 4	1.06
cg25935697	132141	IQCF1	IQ motif containing F1	1.11
cg19769989	51058	ZNF691	zinc finger protein 691	1.11

[1] Linear regression model does not include surrogate variables.

Supplementary Table S8. CpGs differentially methylated at FDR < 0.05 in island-related and promoter regions[1].

Site label	Gene ID	Gene Symbol	Description	Fold Change
**Island**				
cg15613340	26167	PCDHB5	protocadherin beta 5	0.81
cg09171355	81848	SPRY4	sprouty RTK signaling antagonist 4	1.06
cg00699321	5799	PTPRN2	protein tyrosine phosphatase receptor type N2	1.09
cg19769989	51058	ZNF691	zinc finger protein 691	1.11
cg02430797	55753	OGDHL	oxoglutarate dehydrogenase L	1.17
**North Shelf**				
cg18322025	253769	WDR27	WD repeat domain 27	0.78
cg25727554	3833	KIFC1	kinesin family member C1	0.94
cg09072576	54584	GNB1L	G protein subunit beta 1 like	1.04
cg08121523	25885	POLR1A	POLR1A	1.04
cg04649441	10910	SUGT1	SGT1 homolog, MIS12 kinetochore complex assembly cochaperone	1.05
cg18034859	85366	MYLK2	myosin light chain kinase 2	1.07
cg07009570	56122	PCDHB14	protocadherin beta 14	1.15
**North Shore Shore**				
cg26169421	442028	LOC442028	uncharacterized LOC442028	0.95
cg21169617	25822	DNAJB5	DnaJ heat shock protein family (Hsp40) member B5	0.95
cg06116255	6656	SOX1	SRY-box transcription factor 1	1.05
cg07994487	9742	IFT140	intraflagellar transport 140	1.06
**South Shelf**				
cg07190921	3483	IGFALS	insulin like growth factor binding protein acid labile subunit	0.91
cg08489478	118813	ZFYVE27	zinc finger FYVE-type containing 27	0.94
cg17956832	114881	OSBPL7	oxysterol binding protein like 7	0.95
cg16336066	79789	CLMN	calmin	1.05
cg15412087	220323	OAF	out at first homolog	1.07
**South Shore**				
cg25026992	56129	PCDHB7	protocadherin beta 7	0.81
cg23245933	57521	RPTOR	regulatory associated protein of MTOR complex 1	0.94
cg06827256	64746	ACBD3	acyl-CoA binding domain containing 3	0.94
cg15381410	4232	MEST	mesoderm specific transcript	1.04
cg12562418	5919	RARRES2	retinoic acid receptor responder 2	1.04
cg02103875	83450	DRC3	dynein regulatory complex subunit 3	1.09
**Open Sea**				

cg23229110	4921	DDR2	discoidin domain receptor tyrosine kinase 2	1.07
cg07114544	9423	NTN1	netrin 1	1.07
cg08130814	79648	MCPH1	microcephalin 1	1.09
cg25935697	132141	IQCF1	IQ motif containing F1	1.11
**Promoter**				
cg21169617	25822	DNAJB5	DnaJ Heat Shock Protein Family (Hsp40) Member B5	0.95
**Islands in Promoter**				
cg26752888	252969	NEIL2	Nei like DNA glycosylase 2	0.89
cg16720650	100507266	STX18-AS1	STX18 antisense RNA 1	0.91
cg05201673	4722	NDUFS3	NADH:ubiquinone oxidoreductase core subunit S3	0.93
cg17452713	80818	ZNF436	zinc finger protein 436	0.94
cg13342441	7040	TGFB1	transforming growth factor beta 1	0.94
cg01172961	100529261	CHURC1-FNTB	churchill domain containing 1 and farnesyltransferase, CAAX box, beta	0.95
cg00846580	64430	PCNX4	pecanex-like protein 4	1.09
cg22076160	2786	GNG4	guanine nucleotide binding-protein gamma subunit 4	1.15

[1] Linear regression model does not include surrogate variables.

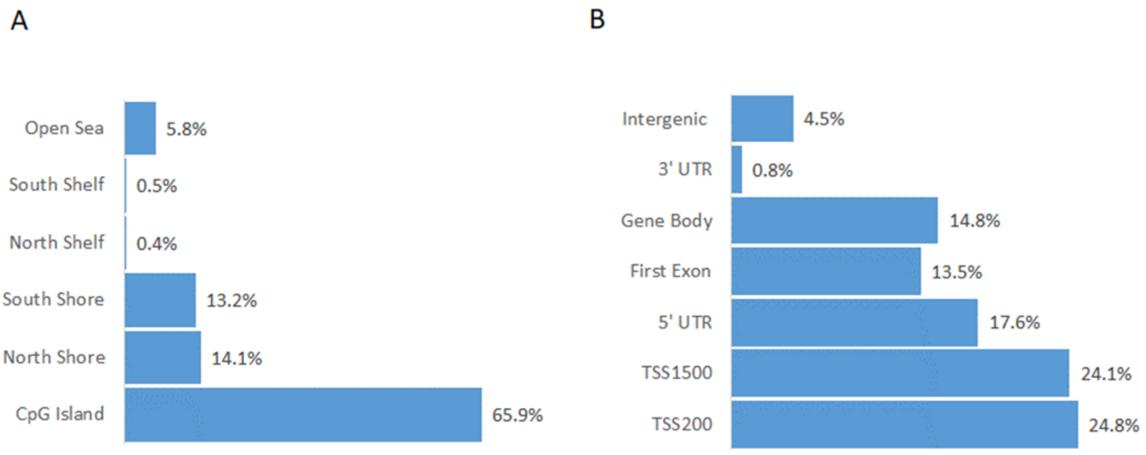

Supplementary Figure 1. Overlap of promoter with gene- or island-related regions. Bar graph depicts the proportion of: (a) coverage of the promoter by CpGs mapping to island-related regions and (b) genic/intergenic regions.

Reference

1. Storey, J.D.; Tibshirani, R. Statistical significance for genomewide studies. *Proc. Natl. Acad. Sci. USA* **2003**, *100*, 9440–9445, doi:10.1073/pnas.1530509100.
